# Using Survival Analysis to Identify Risk Factors for Treatment Interruption among New and Retreatment Tuberculosis Patients in Kenya

**DOI:** 10.1371/journal.pone.0164172

**Published:** 2016-10-05

**Authors:** Enos O. Masini, Omar Mansour, Clare E. Speer, Vittorio Addona, Christy L. Hanson, Joseph K. Sitienei, Hillary K. Kipruto, Martin Muhingo Githiomi, Brenda Nyambura Mungai

**Affiliations:** 1 Ministry of Health, Nairobi, Kenya; 2 Macalester College, St. Paul, Minnesota, United States of America; 3 World Health Organization, Nairobi, Kenya; 4 Centre for Health Solutions, Nairobi, Kenya; Médecins Sans Frontières (MSF), INDIA

## Abstract

Despite high tuberculosis (TB) treatment success rate, treatment adherence is one of the major obstacles to tuberculosis control in Kenya. Our objective was to identify patient-related factors that were associated with time to TB treatment interruption and the geographic distribution of the risk of treatment interruption by county. Data of new and retreatment patients registered in TIBU, a Kenyan national case-based electronic data recording system, between 2013 and 2014 was obtained. Kaplan-Meier curves and log rank tests were used to assess the adherence patterns. Mixed-effects Cox proportional hazards modeling was used for multivariate analysis. Records from 90,170 patients were included in the study. The cumulative incidence of treatment interruption was 4.5% for new patients, and 8.5% for retreatment patients. The risk of treatment interruption was highest during the intensive phase of treatment. Having previously been lost to follow-up was the greatest independent risk factor for treatment interruption (HR: 4.79 [3.99, 5.75]), followed by being HIV-positive not on ART (HR: 1.96 [1.70, 2.26]) and TB relapse (HR: 1.70 [1.44, 2.00]). Male and underweight patients had high risks of treatment interruption (HR: 1.46 [1.35, 1.58]; 1.11 [1.03, 1.20], respectively). High rates of treatment interruption were observed in counties in the central part of Kenya while counties in the northeast had the lowest risk of treatment interruption. A better understanding of treatment interruption risk factors is necessary to improve adherence to treatment. Interventions should focus on patients during the intensive phase, patients who have previously been lost to follow-up, and promotion of integrated TB and HIV services among public and private facilities.

## Background

Kenya had an 88.3% treatment success rate in 2014 among new and relapse cases of tuberculosis (TB). Ninety-five percent of TB patients knew their HIV status, and 87% of HIV-positive TB patients were on antiretroviral therapy (ART) [1]. Although these rates are continuing to improve, TB is a leading cause of death and morbidity in Kenya. It is still considered a “high burden” country, with approximately 88,000 new cases of TB in 2014. There were 9,400 deaths among the TB-infected population and 8,100 deaths among those infected with both TB and HIV [2]. With a national strategic plan and improving methods of data collection, Kenya is taking steps to reduce rates of infection and death from TB. Nevertheless, a significant population of TB patients in Kenya is lost to follow-up at some point during treatment.

This means that such patients do not complete the full regimen necessary to cure them of the disease.

It is worth noting that the loss to follow-up rate has been declining in the last decade from 10% in 2005–2006 to 5% in 2014 [3,4].

Several previous studies have attempted to identify major factors that affect adherence to treatment among TB patients. Structural factors including poverty and gender, patients’ beliefs and attitudes towards treatment, the social environment, and the accessibility of health care services are among the most important factors [5–7]. A study conducted in Nairobi between 2006 and 2008 found that treatment interruption was observed most frequently within the first couple of months of treatment, which is the most intensive period. In addition, the study found higher rates of treatment interruption among HIV-positive patients, those who suffer from alcohol dependence, those with a lack of knowledge about TB treatment, and low-income patients [8].

TIBU is a nationwide electronic database created by Kenya’s National Tuberculosis, Leprosy and Lung Disease Program (NTLD) and is the first such data recording system in the region. TIBU is the Swahili word for *treat*, and is an acronym for Treatment Information from Basic Unit. The system was launched in September 2012. It has been used by Kenya’s Ministry of Health to retrospectively input data from before 2013 and for live capture of all patients diagnosed from 2013 onward. TIBU’s purpose is to continually update these electronic health records to allow for immediate analyses of success in disease treatment and prevention. It has yielded a robust database with comprehensive patient parameters over the last few years. Detailed information about TIBU can be accessed at http://nltp.co.ke/the-tibu-initiative/ [9].

In logistic regression, the response variable is a binary outcome. However, the ambitious target of ending the TB epidemic by 2030 proposed by the Sustainable Development Goals of the United Nations warrants a more granular analysis of TB treatment interruption [10]. Survival analytic techniques are used to study the time until an event occurs. This allows us to treat as right-censored those observations which result in an outcome other than the endpoint of interest. That is, observations for which the time to event is incomplete. For example, patients could move to another country or transfer from one reporting system to another. Furthermore, survival analytic techniques have been commonly used to investigate the association between time to a certain event and other factors in public health studies, and not predominantly time to death [11]. This study therefore uses survival analysis to examine the time to treatment interruption and its relationship to other patient-related factors in order to help guide future research and policy. In addition to providing this new approach to the problem of treatment interruption, this is the first study of TB in Kenya to compute a national risk of treatment interruption as well as county-specific risks.

## Data and Methods

### Study population

Data recorded in TIBU from June 2013 to June 2014 of all cases, including smear positive and smear negative pulmonary (PTB) and extrapulmonary (EPTB), were considered for this analysis (n = 91,049). Most patient-level covariates available in TIBU were obtained and recategorized for the purpose of this analysis: sex, age, patient type, body mass index (BMI), HIV status, nutritional support, sector, directly observed therapy (DOT), and the county where patients received care. The covariates included in the study represent information that was collected and recorded as a single baseline entry when a patient is diagnosed. Due to the newness of TIBU, most patients’ information does not get updated through the course of treatment.

The BMI of patients who have a BMI below 12.5 kg/m^2^ and above 42.5 kg/m^2^ was classified as unknown due to an apparent recording error. Patients were categorized into four groups based on their BMI: underweight (<18.5 kg/m^2^), normal (18.5–25 kg/m^2^), overweight (25–30 kg/m^2^), and obese (≥30 kg/m^2^).

Patient type has two levels: new patients and retreatment patients. In Kenya, patients who have never been treated for TB or have taken anti-TB drugs for less than one month are considered new patients. On the other hand, patients who have previously been treated for TB for more than one month are considered retreatment patients. Retreatment patients are divided into four different groups in TIBU: relapse, treatment after failure, treatment after loss to follow-up, and other retreatment patients. Case definitions for retreatment were adopted from Definitions and Reporting Framework for Tuberculosis by World Health Organization [12]. The Kenya TB treatment guidelines state that all new TB patients should receive the 6-month regimen and retreatment patients receive the 8-month regimen. The 8-month regimen consists of two months of Streptomycin (S), Rifampicin (R), Isoniazid (H), Pyrazinamide (Z), Ethambutol (E) followed by one month of RHZE and then five months of RHE (abbreviated 2SRHZE/1RHZE/5RHE). The first three months of the 8-month regimen are referred to as the intensive phase while the remaining five months are referred to as the continuous phase. The 6-month regimen consists of taking RHZE for the first two months and then RH for the following four months (abbreviated 2RHZE/4RH). The first two months of the 6-month regimen are referred to as the intensive phase while the remaining four months are referred to as the continuous phase. Patients were considered lost to follow-up if they did not start treatment after diagnosis or their treatment was interrupted for two consecutive months or more.

HIV status was categorized as: HIV-negative, HIV-positive on ART, or HIV-positive not on ART. Nutritional support was divided into four categories: caloric support (food support), non-caloric support (counseling and micronutrients), caloric and non-caloric support, and no nutritional support. Nutritional support is allocated to patients loosely based on their BMI.NTLD policy states that patients who have a BMI below 18.5 should, at the very least, receive caloric support. However, the BMI cutoff for caloric support is occasionally lowered to 16 when resources are limited. However, some private facilities and non-governmental organizations provide nutritional support to all of their patients. Sector refers to the type of health facility where the patient was diagnosed and entered into TIBU. It includes public facilities, private facilities, prisons, and faith-based facilities. DOT was divided into three categories: family-based (household member, relative, or friend), community volunteer (CV), or healthcare worker (HCW).

### Treatment outcomes and censoring

TB patients were categorized into six different groups according to their treatment outcome in TIBU: cured, treatment completed, treatment failed, died, lost to follow-up, and transferred out. For the purposes of our analysis, only the times for patients who were lost to follow-up were treated as exact times; for all other outcomes, times were regarded as right-censored.

Treatment duration was determined by calculating the difference in days between date of treatment initiation and date of treatment termination. New cases for which the observed treatment duration exceeded 180 days were excluded from this analysis because of apparent recording issues and the inability to estimate the timing of treatment interruption. Similarly, retreatment cases for which the observed treatment duration exceeded 240 days were excluded.

### Statistical analysis

All analyses were conducted using the R programming language [13]. Kaplan-Meier curves were used to display the probability of treatment interruption over time for each risk factor, the predictors included in TIBU. For brevity, Kaplan-Meier graphs that yielded insignificant log-rank test p-values at the 5% level were omitted, but available upon request from the authors. Thus, the survival curves included represent each risk factor which showed a statistically significant association with time to treatment interruption. In order to explore continuous covariates and multivariate models, we also constructed Cox proportional hazards models for time to treatment interruption. Our Cox models were mixed-effects models in that they incorporated both fixed effects, and accounted for the geographic region of Kenya via a random effects component. We present the results of our Cox models by reporting hazard ratios (HR) and the corresponding 95% confidence interval, along with Wald test p-values. To visualize the random effects component, we present a map of Kenya with each county colored according to the magnitude of its random effects coefficient. The proportionality of hazards assumption made in our Cox models was examined for all risk factors. None of the risk factors were deemed to violate the proportional hazards assumption. Only complete cases were included in the Cox proportional hazards models (cases with missing values were excluded from the analysis).

The study was approved by the Kenyan Ministry of Health, Nairobi, Kenya. No individual identifiers were reviewed as part of this analysis.

## Results

Records from 90,170 patients were included in the analysis. Approximately, 90% of the patients were new, 60% were male, 50% were underweight, and 35% were HIV-positive. Among HIV-positive patients, 87% were on ART. More than 50% of patients were between 15 and 36 years old, less than 10% under the age of 15, and 6% were above 62 years old. Furthermore, the majority of patients received care either at a public or private facility (78% and 20%, respectively). Most patients had family-based DOT while a small proportion of patients received DOT from healthcare workers (87.5% and 11.7%, respectively). Thirteen percent of patients did not receive any form of nutritional support, 63.6% received only non-caloric support, 7% received only caloric support, and 16.5% received both (Table 1).

**Table 1:** Characteristics of TB population in TIBU.

Risk Factor	Cured N (%)	Completed N (%)	Failed N (%)	Died N (%)	lost to follow-up N (%)	Transferred out N (%)	Total N (%)
**Patient type**							
New	29,045 (35.6)	41,544 (51.0)	368 (0.5)	4,985 (6.1)	3,568 (4.4)	1,991 (2.4)	81,600 (90.5)
Relapse	2,357 (38.8)	2,520 (41.5)	78 (1.3)	520 (8.6)	386 (6.4)	212 (3.4)	6,091 (6.8)
After failure	174 (74.0)	15 (6.4)	18 (7.7)	9 (3.8)	16 (6.8)	3 (1.3)	240 (0.3)
After loss to follow-up	355 (27.6)	507 (39.5)	10 (0.8)	109 (8.5)	253 (19.7)	50 (3.9)	1,288 (1.4)
Other retreatment	1 (0.1)	766 (80.9)	0 (0.0)	113 (11.9)	42 (4.4)	25 (2.7)	951 (1.0)
**TB type**							
Pulmonary	32,218 (43.1)	32,166 (43.0)	481 (0.6)	4,431 (5.9)	3,588 (4.8)	1,876 (2.6)	74,861 (82.2)
Extrapulmonary	8 (0.1)	13,650 (84.5)	0 (0.0)	1,344 (8.3)	711 (4.4)	445 (2.7)	16,188 (17.8)
**Sex**							
Female	11,500 (31.6)	20,125 (55.3)	136 (0.4)	2,307 (6.3)	1,359 (3.7)	934 (2.7)	36,416 (40.0)
Male	20,726 (38.0)	25,691 (47.0)	345 (0.6)	3,468 (6.4)	2,940 (5.4)	1,387 (2.6)	54,633 (60.0)
**Age**							
Below 15	805 (9.3)	6,921 (80.3)	11 (0.1)	416 (4.8)	288 (3.3)	178 (2.2)	5,514 (9.5)
Between 15 and 27	11,217 (47.0)	9,766 (40.9)	106 (0.4)	785 (3.3)	1,222 (5.1)	761 (3.3)	23,884 (26.2)
Between 28 and 36	9,669 (40.2)	10,886 (45.2)	158 (0.7)	1,429 (5.9)	1,286 (5.3)	651 (2.7)	24,104 (26.5)
Between 37 and 47	6,106 (34.4)	8,990 (50.6)	119 (0.7)	1,325 (7.5)	809 (4.6)	413 (2.2)	17,789 (19.5)
Between 48 and 62	3,311 (29.9)	6,005 (54.1)	66 (0.6)	1,044 (9.4)	456 (4.1)	207 (1.9)	11,115 (12.2)
Above 62	1,114 (20.3)	3,244 (59.0)	21 (0.4)	755 (14.1)	233 (4.2)	111 (2.0)	5,514 (6.1)
**BMI**							
Normal	12,407 (37.8)	16,237 (49.5)	141 (0.4)	1,658 (5.1)	1,463 (4.5)	885 (2.7)	32,833 (44.4)
Underweight	14,939 (41.0)	15,967 (43.8)	271 (0.7)	2,568 (7.0)	1,843 (5.1)	887 (2.4)	36,513 (49.4)
Overweight	891 (25.5)	2,222 (63.7)	5 (0.1)	189 (5.4)	100 (2.9)	83 (2.4)	3,499 (4.7)
Obese	267 (24.7)	715 (66.3)	3 (0.3)	39 (3.6)	34 (3.2)	21 (1.9)	1,082 (1.5)
**HIV status**							
Negative	23,006 (42.2)	25,400 (46.6)	325 (0.6)	2,100 (3.9)	2,478 (4.5)	1,230 (2.2)	54,624 (60.0)
Positive on ART	6,870 (25.0)	15,832 (57.6)	126 (0.5)	2,835 (10.3)	1,116 (4.1)	728 (2.5)	27,530 (30.2)
Positive not on ART	938 (23.4)	2,018 (50.2)	17 (0.4)	537 (13.4)	324 (8.1)	183 (4.5)	4,023 (4.4)
Not tested	1,412 (29.1)	2,566 (52.9)	13 (0.3)	303 (6.2)	381 (7.8)	180 (3.7)	4,872 (5.4)
**Sector**							
Public	26,113 (36.8)	34,697 (48.9)	404 (0.6)	4,645 (6.5)	3,477 (4.9)	1,685 (2.3)	71,118 (78.1)
Private	5,524 (30.3)	10,311 (56.6)	69 (0.4)	1,048 (5.6)	758 (4.2)	507 (2.9)	18,248 (20.0)
Faith-based	77 (30.6)	138 (54.8)	4 (1.6)	20 (7.9)	8 (3.2)	5 (1.9)	253 (0.3)
Prisons	512 (35.9)	670 (46.9)	4 (0.3)	62 (4.3)	56 (3.9)	124 (8.7)	1,430 (1.6)
**DOT**							
Family-based	28,125 (35.4)	40,546 (51.0)	371 (0.5)	4,955 (6.2)	3,612 (4.5)	1,939 (2.4)	79,641 (87.5)
CV	258 (37.2)	324 (46.7)	2 (0.3)	62 (8.9)	36 (5.2)	12 (1.7)	695 (0.8)
HCW	3,827 (36.0)	4,917 (46.3)	108 (1.0)	753 (7.1)	646 (6.1)	367 (3.5)	10,655 (11.7)
**Nutritional support**							
No support	3,775 (33.7)	5,889 (52.6)	39 (0.3)	522 (4.7)	596 (5.3)	371 (3.4)	11,231 (12.9)
Non-caloric	20,016 (36.1)	27,956 (50.4)	288 (0.5)	3,286 (5.9)	2,556 (4.6)	1,375 (2.5)	55,532 (63.6)
Caloric	2,122 (35.1)	3,008 (49.7)	31 (0.5)	435 (7.2)	287 (4.7)	165 (2.8)	6,057 (7.0)
Caloric and non-caloric	5,066 (35.1)	7,071 (49.0)	103 (0.7)	1,274 (8.8)	624 (4.3)	301 (2.1)	14,457 (16.5)

CV: community volunteer; HCW: healthcare worker.

Treatment interruption rate was highest among those who had previously been lost to follow-up (19.7%). In addition, treatment interruption rate was higher among males compared to females (5.4% and 3.7%, respectively). HIV-positive patients not on ART and those who were not tested for HIV had higher treatment interruption rates than those who were HIV-negative or HIV-positive on ART (Table 1).

**Fig 1:**
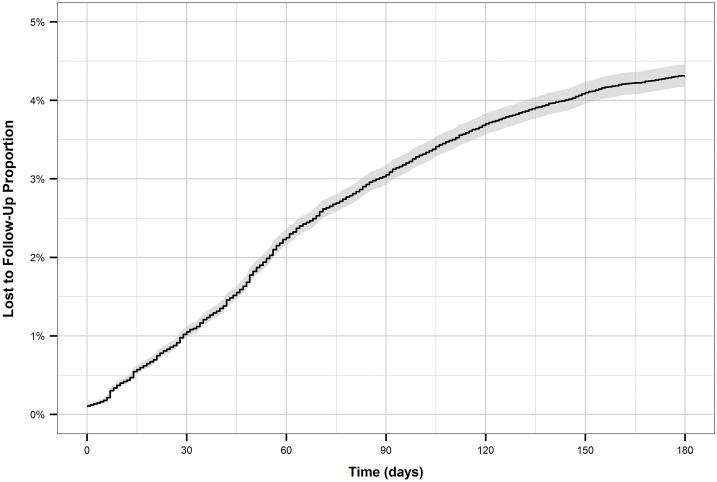
Cumulative incidence of loss to follow-up for new patients.

**Fig 2:**
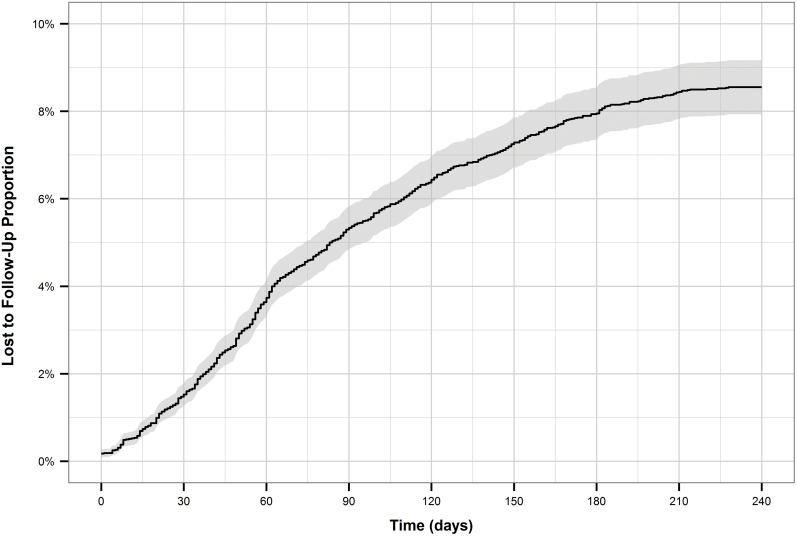
Cumulative incidence of loss to follow-up for retreatment patients.

**Fig 3:**
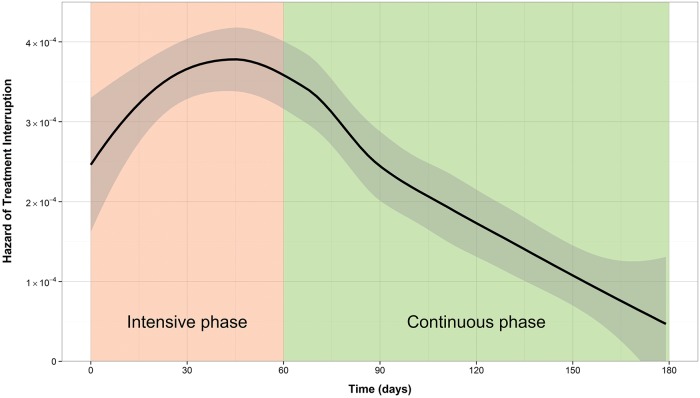
Hazard function of loss to follow-up for new patients.

**Fig 4:**
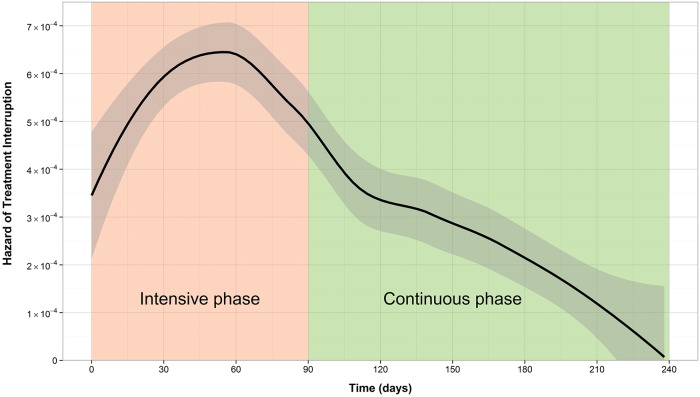
Hazard function of loss to follow-up for retreatment patients.

The cumulative incidence of treatment interruption was 4.5% for new patients, and 8.5% for retreatment patients. For both patient types, the cumulative incidence increased more rapidly during the first two to three months of treatment (Figs 1 and 2). This is reflected by the hazard function plots (Figs 3 and 4). The hazard of treatment interruption was highest for both patient types during the intensive phase. The hazard then decreased notably for both groups over the course of the continuous phase. It is important to note that the initial hazard of treatment interruption for retreatment patients increased more rapidly during the intensive phases compared to new patients.

**Fig 5:**
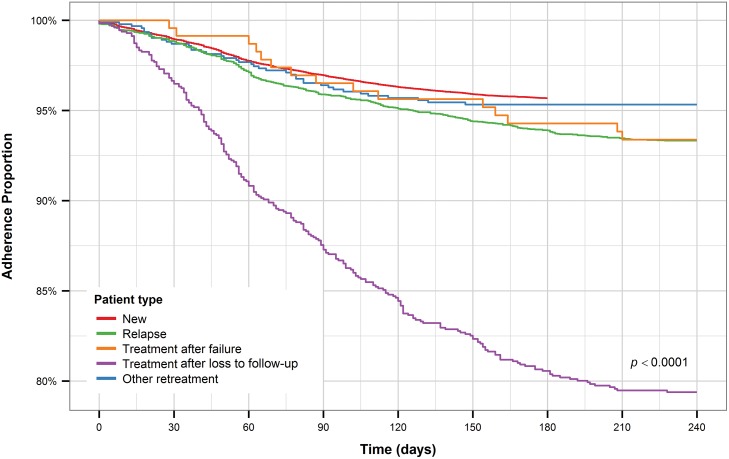
Kaplan-Meier plot for TB patient type.

**Fig 6:**
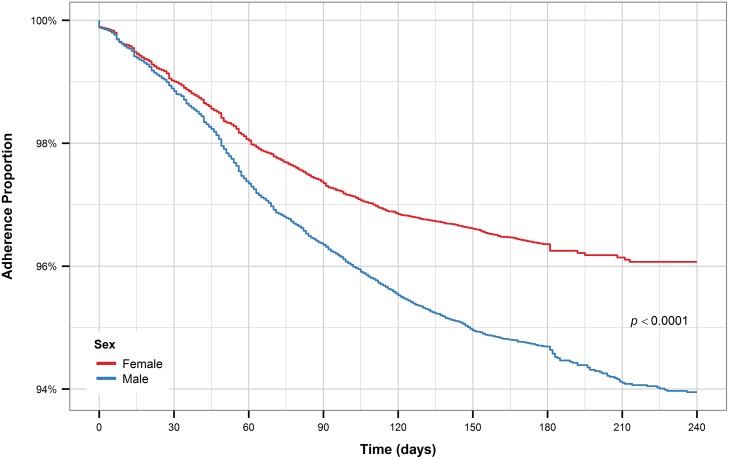
Kaplan-Meier plot for sex (all patients).

Retreatment after loss to follow-up patients had the shortest time to treatment interruption, with all other groups exhibiting similar times to treatment interruption (Fig 5). Male TB patients had a significantly shorter time to treatment interruption compared to female TB patients (Fig 6). The pattern holds for new and retreatment patients combined and separately.

**Fig 7:**
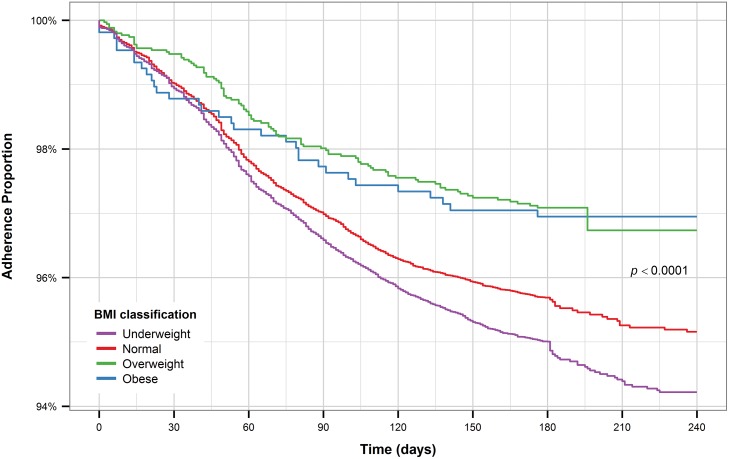
Kaplan-Meier plot for BMI (all patients).

**Fig 8:**
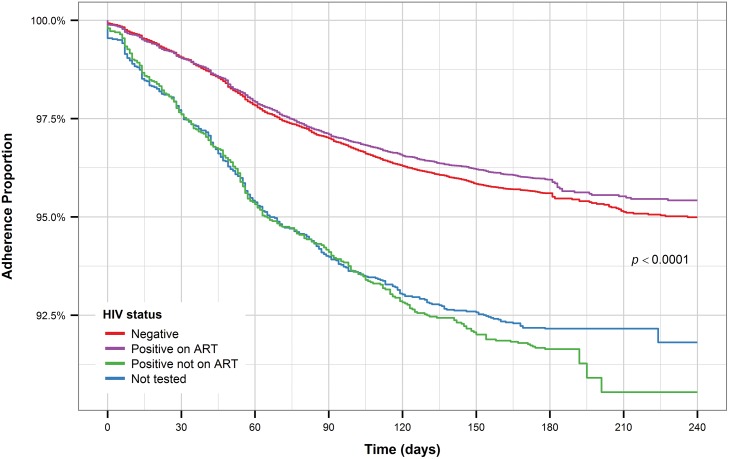
Kaplan-Meier plot for HIV status (all patients).

Underweight patients have the shortest time to treatment interruption compared to patients with normal BMI while overweight and obese patients have the longest time to treatment interruption (Fig 7). HIV patients who are on ART and HIV-negative patients have a noticeably longer time to treatment interruption compared to HIV-positive patients not on ART and patients who were not tested for HIV (Fig 8).

**Fig 9:**
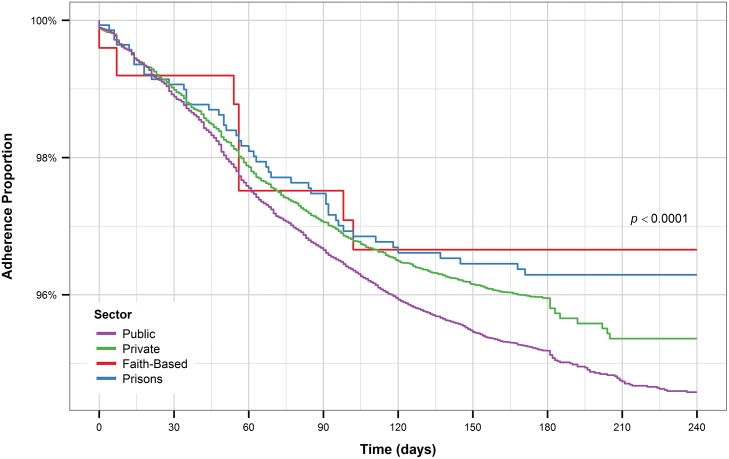
Kaplan-Meier plot for sector (all patients).

**Fig 10:**
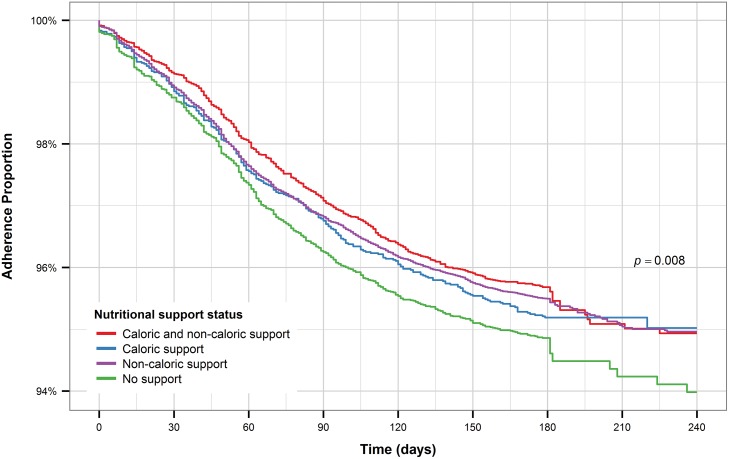
Kaplan-Meier plot for nutritional support (all patients).

Patients who received care at faith-based health facilities had the longest time to treatment interruption compared to the other facility types (Fig 9). On the other hand, patients who received care at public facilities had the shortest time to treatment interruption. Patients who received caloric and non-caloric support combined, along with those receiving only non-caloric support had the longest time to treatment interruption while patients who received only caloric support had the shortest time to treatment interruption, even compared to patients who received no form of nutritional support (Fig 10).

**Fig 11:**
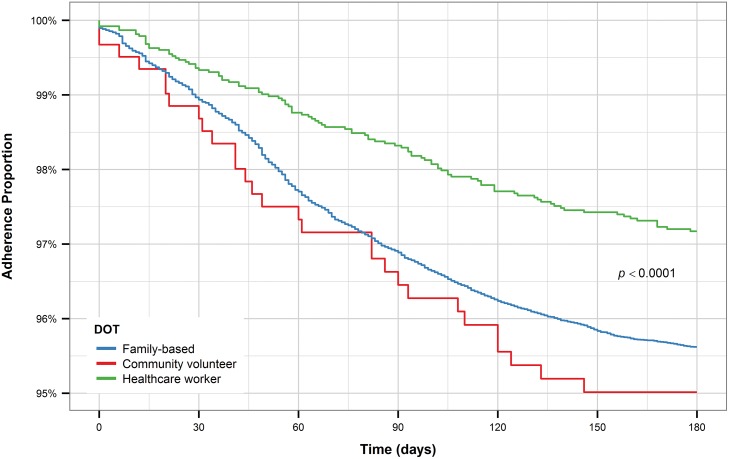
Kaplan-Meier plot for DOT (new patients).

**Fig 12:**
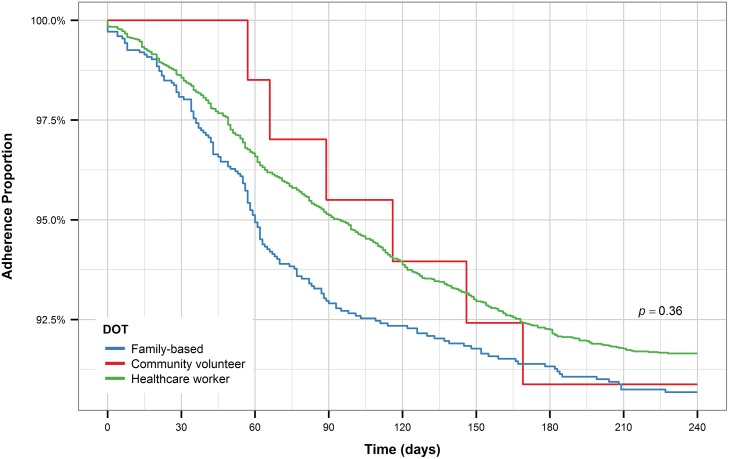
Kaplan-Meier plot for DOT (retreatment patients).

For both new and retreatment patients, individuals who received DOT from healthcare workers had longer time to treatment interruption than patients who had family-based DOT (Figs 11 and 12). While new patients who received DOT for community volunteers had a shorter time to treatment interruption compared to patients who received either of the other two forms of DOT, retreatment patients who received DOT from community volunteers had a similar time to treatment interruption as retreatment patients who received DOT from healthcare workers and longer time to treatment interruption compared to patients who received family-based DOT. However, this pattern is most likely observed due to the small number of patients who received DOT from community volunteers, especially among retreatment patients.

The results of the mixed-effects Cox survival model are summarized in Table 2. Retreatment patients were more likely to be lost to follow-up than new patients. In particular, those who had previously been lost to follow-up and relapse patients were more likely to be lost to follow-up than new patients [HR: 4.79, HR: 1.70, respectively]. There was no difference in the risk of treatment interruption between patient with EPTB and with PTB. Males were 1.5 times as likely to be lost to follow-up as females. Children (<15) were notably less likely to be lost to follow-up compared to young adults (15–27) [HR: 0.42]. Similarly, middle-aged (37–47; 48–62) and old patients (≥62) were less likely to be lost to follow-up compared to young adults [HR: 0.83, HR: 0.74, HR: 0.82, respectively]. The difference in risk of treatment interruption between adults (28–36) and young adults was not statistically significant (Table 2).

**Table 2:** Survival models results for risk factors associated with treatment interruption.

	Unadjusted Model*		Adjusted Model^†^	
Risk Factor	HR (95% CI)	*p*-value	HR (95% CI)	*p*-value
**Patient type**				
New	reference		reference	
Relapse	1.42 (1.27–1.58)	<0.0001	1.70 (1.44–2.00)	<0.0001
After failure	1.42 (0.86–2.36)	0.17	1.51 (0.87–2.65)	0.15
After loss to follow-up	4.52 (3.96–5.16)	<0.0001	4.79 (3.99–5.75)	<0.0001
Other retreatment	1.00 (0.73–1.36)	0.99	1.29 (0.87–1.90)	0.20
**TB type**				
Pulmonary	reference		reference	
Extrapulmonary	0.90 (0.82–0.96)	0.01	0.90 (0.80–1.00)	0.51
**Sex**				
Female	reference		reference	
Male	1.46 (1.37–1.57)	<0.0001	1.46 (1.35–1.58)	<0.0001
**Age**				
Below 15	0.67 (0.59–0.77)	<0.0001	0.42 (0.32–0.56)	<0.0001
Between 15 and 27	reference		reference	
Between 28 and 36	1.06 (0.98–1.15)	0.13	1.03 (0.94–1.12)	0.58
Between 37 and 47	0.93 (0.85–1.02)	0.13	0.83 (0.74–0.92)	<0.001
Between 48 and 62	0.85 (0.76–0.95)	<0.01	0.74 (0.65–0.84)	<0.0001
Above 62	0.96 (0.83–1.12)	0.63	0.82 (0.69–0.97)	0.023
**BMI**				
Normal	reference		reference	
Underweight	1.16 (1.08–1.24)	<0.0001	1.11 (1.03–1.20)	0.01
Overweight	0.66 (0.54–0.81)	<0.0001	0.79 (0.64–0.98)	0.03
Obese	0.69 (0.49–0.98)	0.04	0.89 (0.62–1.28)	0.52
**HIV status**				
Negative	reference		reference	
Positive on ART	0.94 (0.88–1.02)	0.14	0.98 (0.89–1.07)	0.62
Positive not on ART	1.87 (1.66–2.12)	<0.0001	1.96 (1.70–2.26)	<0.0001
Not tested	1.64 (1.46–1.83)	<0.0001	1.55 (1.33–1.81)	<0.0001
**Sector**				
Public	reference		reference	
Private	0.80 (0.73–0.87)	<0.0001	0.79 (0.71–0.87)	<0.0001
Faith-based	0.80 (0.38–1.55)	0.46	0.60 (0.22–1.61)	0.31
Prisons	0.74 (0.55–0.98)	0.03	0.73 (0.53–1.00)	0.05
**DOT**				
Family-based	reference		reference	
CV	1.13 (0.81–1.58)	0.47	0.98 (0.66–1.44)	0.91
HCW	1.43 (1.31–1.56)	<0.0001	0.85 (0.73–0.99)	0.03
**Nutritional support**				
No support	reference		reference	
Non-caloric	0.98 (0.89–1.08)	0.72	1.09 (0.96–1.23)	0.17
Caloric	1.03 (0.89–1.20)	0.66	1.21 (1.02–1.44)	0.03
Caloric and non-caloric	1.00 (0.89–1.13)	0.97	1.08 (0.93–1.25)	0.30

*Univariate analysis,

^†^Multivariate analysis.

HR: hazard ratio; CI: confidence interval; CV: community volunteer; HCW: healthcare worker.

Underweight patients were more likely to be lost to follow-up and overweight patients were less likely to be lost to follow-up compared to those classified as having normal BMI [HR: 1.11, HR: 0.79, respectively]. The difference in risk between obese patients and normal patients was not statistically significant (Table 2). Additionally, the difference between HIV-positive patients on ART and HIV-negative patients was not statistically significant. On the other hand, HIV-positive patients not on ART and those who had not been tested had a particularly high risk of treatment interruption compared to HIV-negative patients [HR: 1.96, HR: 1.55, respectively]. Patients who received care at a private facility were less likely to be lost to follow-up compared to patients who received treatment at a public facility [HR: 0.79]. The difference in risk between patients who received care at a faith-based facility or prison and patients who received care at a public facility was not statistically significant. Patients who received DOT from healthcare workers were less likely to be lost to follow-up compared to patients who received family-based DOT [HR: 0.85]. However, the difference in the risk between patients who received DOT from community volunteers and those who received family-based DOT was not statistically significant. Patients who received caloric nutritional support were more likely to be lost to follow-up compared to those who received no support [HR: 1.21]. There was no difference in risk between the other levels of nutritional support and having no nutritional support.

**Fig 13:**
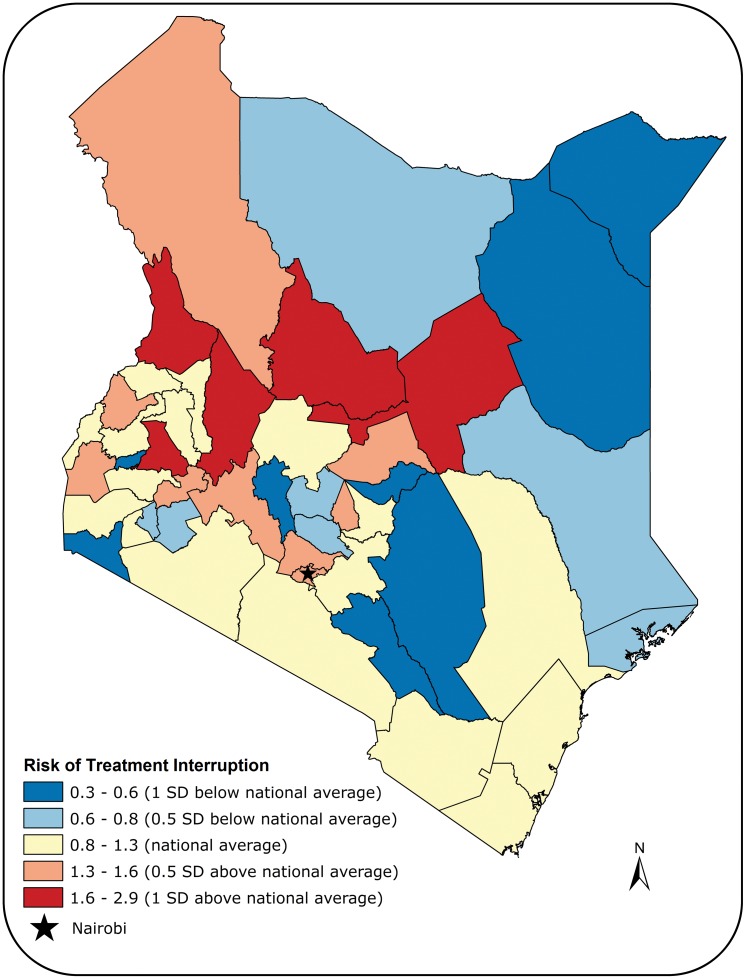
Map showing the geographic distribution of the risk of default by county.

Three clusters of varying risks of treatment interruption were identified at the county-level (Fig 13). Most counties with high risk of treatment interruption, compared to the national average, were located in the central part of Kenya. The counties with the highest risk of treatment interruption were Samburu, West Pokot, and Baringo [HR: 2.9, 2.4, and 2.3, respectively]. Counties in the northeast had lower risk of treatment interruption compared to the national average. Nyandarua, Wajir, and Mandera had the lowest risk of treatment interruption [HR: 0.5, 0.4, and 0.3, respectively]. The risk of treatment interruption was close to the national average for the majority of counties in the southwest. It is worth noting that Nairobi had a treatment interruption risk of 1.4.

## Discussion

By utilizing survival analytic techniques that account for censoring, this study was able to identify several patient-level risk factors that are associated with TB treatment interruption in Kenya. To produce more reliable estimates, we accounted for geographic variability of treatment default rates via a random effects component. The study found that approximately 4.5% of new patients in Kenya were lost to follow-up, compared with 8.5% of retreatment patients. In addition, the hazard of default was at its highest during the intensive phase of treatment for both new and retreatment patients. By analyzing a comprehensive national TB database, our results can help plan effective future interventions that address TB treatment interruption in Kenya and in similar settings.

Patients who had previously been lost to follow-up from anti-TB treatment had a significantly high risk of being lost to follow-up again [8,14]. This demonstrates that those patients require special attention once they are captured by the healthcare system to reduce their high risk of treatment interruption. Considering that patients who received DOT from healthcare workers were less likely to be lost to follow-up compared to those who received family-based DOT, patients who have previously been lost to follow-up should be assigned to healthcare workers for their DOT. On the other hand, there was no difference in the association between the other two types of DOT, the community volunteering program and the family-based DOT, and treatment adherence. It is important to note that before adjusting for other patient covariates, patients who received DOT from healthcare workers were more likely to be lost to follow-up compared to patients who received family-based DOT. This is due to the fact that retreatment patients were more likely to receive DOT from healthcare workers compared to new patients (80% to 5%, respectively). Nevertheless, more than 25% of those who had previously been lost to follow-up received family-based DOT or DOT from community volunteers. The NTLD should direct more resources toward training additional healthcare workers or look into measures that could improve the effectiveness of family-based DOT and the community volunteering program. HIV-negative patients and HIV-positive patients on ART had approximately the same risk of treatment interruption from TB treatment. Conversely, TB patients who were HIV-positive but not on ART and those who had not been tested for HIV were more likely to be lost to follow-up than those who were HIV-negative. Considering that 95% of TB patients know their HIV status and more than 87% of HIV-positive patients receive ART, those who were not on ART or did not get tested represent a small proportion of TB patients in Kenya. This speaks to the success of the NTLD policy of having integrated TB and HIV services. An example of this is the provider-initiated HIV testing and counseling (PITC) in which all patients are offered services unless they decide to opt-out [15]. Furthermore, these results confirm the success of ART in keeping HIV-positive patients relatively healthy and improving TB treatment outcome [16,17].

Men had a notably higher risk for treatment interruption than women. This trend has been observed in studies of TB in other countries and has been attributed to men having higher employment rates and being less likely to seek healthcare after the onset of possible TB symptoms [5,14,18]. In Kenya, men are more likely to be employed compared to women, and it has been observed that TB patients who are employed have work-related issues that influence treatment adherence such as difficulty in obtaining sick leave for treatment and fear of losing work or dismissal [18–20]. Similarly, the age groups with the highest risk of treatment interruption were patients of working age, particularly young adults. The groups with the lowest risk of treatment interruption compared to young adults were children and seniors, those least likely to be following a work schedule. A common suggestion to address default among working patients is to change clinics’ operating hours so that working patients can access their services at more convenient times, while another possibility would be to provide DOT at the workplace [21,22].

While our results indicate that patients who received care at private health facilities were less likely to be lost to follow-up, they do not necessarily reflect a lower quality of care provided by the public sector. In Kenya, TB care is provided for free by the public sector while patients have to cover their treatment costs at private facilities [23]. This could mean that the socio-economic status or other inherent characteristics of those who seek care in the private sector are different than those who seek care in the public sector. Additionally, all health facilities are required to report newly detected TB cases to the NTLD. However, not all private facilities report their cases. Those who report cases are, simply by the nature of their reporting, showing themselves to be more in compliance with national standards. As such, this finding cannot be extrapolated to represent the entire private sector. The NTLD should continue promoting private–public partnerships to increase the number of private providers integrated into TIBU.

Patients with low BMI had higher risk of treatment interruption than those with normal BMI. This was expected, as poor treatment outcomes are associated with malnourishment. Approximately 50% of TB patients in TIBU were malnourished. One measure to mitigate the effects of malnourishment on treatment adherence is to provide nutritional support. However, most forms of nutritional support were not associated with the risk of treatment interruption. Although our results indicate that caloric support was associated with higher risk of treatment interruption, the underlying relationship is not clear. The evidence on the impact of nutritional support is conflicting [24–29]. Additionally, we found huge variability in the association between nutritional support and the risk of treatment interruption across different counties and sub-populations. The relationship seems to be complex and further research is needed to evaluate the effectiveness of nutritional support in various settings. Overweight patients had a slightly lower risk of treatment interruption compared to those with normal BMI, which could reflect a higher socioeconomic status.

Finally, there was a large amount of variability in the risk of treatment interruption between the different counties in Kenya. Generally, arid and semi-arid regions and counties with nomadic populations, such as Isiolo and Samburu, had the highest risk of treatment interruption. This could be due to limited access to health facilities or the mobile lifestyle of patients in these counties. Low risk of treatment interruption was associated with counties that had small numbers of TB patients, such as Mandera, Wajir, and Garissa. Further research is warranted to understand the factors contributing to the variability in the risk of treatment interruption among counties in Kenya.

This study is not without limitations. TIBU includes numerous observations with recording errors (e.g., invalid treatment initiation and termination dates and missing information). Also, the study did not assess the impact of several known TB risk factors such as smoking and diabetes. We did not have information on income, which might be a confounding factor in our analysis. Additionally, we were not able to acknowledge the time-varying nature of certain covariates since only baseline values were consistently available from TIBU. It will be important to incorporate TB risk factors that are not included in TIBU in future studies. Addressing treatment interruption could lead to a reduction in the transmission of TB as well as the cost of providing repeated treatment.
